# One Health education for criticality on vaccination in teacher training

**DOI:** 10.3389/fpubh.2024.1408965

**Published:** 2024-07-26

**Authors:** Inés Martínez-Pena, Blanca Puig, Araitz Uskola

**Affiliations:** ^1^Faculty of Education, Universidade de Santiago de Compostela (USC), Santiago, Spain; ^2^Department of Didactics of Mathematics and of Experimental and Social Sciences, University of the Basque Country (UPV/EHU), Leioa, Spain

**Keywords:** One Health, vaccines, criticality, critical ignoring, biology education, teacher training

## Abstract

**Introduction:**

Vaccines are the basis of health of our communities since they prevent severe infectious diseases. However vaccination rates continue to decrease due to the spread of misinformation about their side effects, which enhances vaccine hesitancy and puts at risk public health. Introducing vaccines from the One Health approach can help to develop an integral understanding of their role and to apply critical ignorance as part of criticality to avoid vaccine hesitancy and raise trust in science. This paper presents a design on vaccination for secondary-education teacher training developed toward this goal.

**Methods:**

The design presented in this paper draws from previous studies on critical thinking, on vaccine rejection, and the One Health approach on other health issues in Secondary Education. The focus of this design is engaging secondary-education pre-service teachers in the practice of critical ignorance and criticality to assess diverse pieces of information on vaccination from the One Health approach.

**Results:**

This study discusses the design principles and the activities of an original design that aims to provide Secondary Education teachers with some tools to introduce critical ignorance and criticality for addressing misinformation on vaccines by using the One Health approach.

**Discussion:**

If secondary science teachers are going to successfully confront misinformation on vaccination in their science instruction, we need to develop and test designs and approaches that prepare them for this purpose. Critical ignorance plays a central role in managing misinformation; thus, such instruction should engage future teachers in critical evaluation of information on vaccination, as well as in the application of the One Health approach to take responsible actions.

## 1 Introduction

Since their discovery vaccines have contributed to save millions of lives throughout History and allowed the eradication of devastating diseases (1, 2). Recently, the COVID-19 pandemic highlighted the relevance of vaccination. It is estimated that COVID-19 vaccines contributed to save around 1.4 million lives in Europe between December 2020 and March 2023 (3).

Despite the relevance of vaccines to preserve health, there is a concerning growth of vaccine hesitancy among population (4). This is fostered by the quick spread of fallacies and fake-news and increases the likelihood of preventable-disease outbreaks. Education in vaccines is an essential tool to raise awareness about the importance of vaccination and to fight against misinformation. Understanding how vaccines work and why they are important is a complex task that requires considering several dimensions of the problem besides human health. Consequently, vaccine education should integrate approaches that allow the development of a global view of the problem. One Health (OH) is an approach that considers the health of humans, animals, and ecosystems as interdependent, providing a global view of complex health issues, such as vaccination.

Assessing vaccination and managing misinformation also requires the application of critical thinking (CT) skills, especially, critical ignoring (CI) and criticality. CI is the ability to select the information, and avoid low-quality information to control own’s informational environment (5). This will help citizens not only to develop their own opinion based on scientific evidence, and to differentiate evidence-based information from pseudoscientific claims, but also to take actions according to their opinion, putting CT in practice by committing to individual and collective actions, as the concept of criticality points out.

The literature review showed that most approaches that introduce vaccination in health education limit to focus on human health, without considering environmental factors that affect this issue (6). This study seeks to make a relevant contribution on this and is in line with current trends regarding the understanding of health as a global issue that not only involves humans, but also the health of animals, plants and ecosystems, a view that is coherent with the OH approach (7). This design seeks to help teachers to promote an integral OH view of vaccination, and to use teaching strategies for managing information and to make responsible actions.

## 2 Didactical framework

### 2.1 Educational challenges to promote criticality on vaccination

Even though the benefits of vaccination are widely supported by scientific evidence, vaccine hesitancy is a current concern in our society. Vaccine hesitancy was defined by the World Health Organization (WHO) Strategic Advisory Group of Experts (SAGE) on Immunization as:

*“Vaccine hesitancy refers to delay in acceptance or refusal of vaccines despite availability of vaccination services. Vaccine hesitancy is complex and context specific* var*ying across time, place and vaccines. It includes factors such as complacency, convenience and confidence.”* (8) (p. 575).

Vaccine hesitancy includes people who show low or no confidence in vaccines but may support vaccination in certain situations and/or contexts. Anti-vaccine movements are located on one extreme of the continuum of vaccine hesitancy. Anti-vaccine individuals deny the efficacy of vaccines, totally rejecting their use independently on the context and circumstances (9).

One of the main concerns of vaccine-hesitants and anti-vaccine individuals is the safety of vaccines (10). This lack of trust is enhanced by the spread of fallacies with no scientific evidence (e.g., vaccines cause autism, contain compounds that poison us, it is better the natural immunity than the immunity generated by vaccines, mRNA vaccines modify our genome…) (11, 12).

In the post-truth era, vaccine misconceptions and fake news are quickly spread by social media promoting hesitancy (4, 13). This leads to a reduction in vaccine coverage among the population increasing the risk of preventable-disease outbreaks. Diseases that have long ceased to be a problem (e.g., measles) are currently experiencing outbreaks due to undervaccination in developed countries like the US and Europe (14, 15).

This also shows the existence of a reducing level of trust in science. The low trust in science might be enhanced by a wide range of factors, such as complex and abstract scientific vocabulary, low ability to manage the uncertainty inherent to the construction of scientific knowledge, and the lack of knowledge regarding the Nature of Science. Moreover, the spread of fake news affects how people deal with scientific information. Science educators seek to promote trust in science taking into consideration these challenges, as well as misconceptions that have been already identified in the literature on vaccination (12). This design aims to provide teachers with tools to introduce efficient strategies for managing information to avoid misinformation and promote trust in science.

### 2.2 OH education for the practice of criticality in the context of vaccination

Raising awareness about vaccination and reducing hesitation is one of the main goals in health education. Health education is part of the biology curriculum in Secondary Education (12–16 years old) in our country (blinded for review). However, health problems have been introduced as human centered SSIs, without an explicit connection with the environment. In response to this, and according to the new science curriculum (blinded for review) that includes the OH approach, science educators need to promote health education from a systemic perspective aligned with the OH approach to improve teachers’ and students’ understanding of vaccination from an integral view (16–18). This is the approach followed in this design.

A deep understanding of the socio-scientific dimensions of vaccination and their potential to keep the health of a community requires the development of the OH approach. OH claims that the health of humans, animals, and ecosystems (including plants) are closely interdependent (7). Introducing the OH approach to teach vaccination will allow to comprehend the impact at different levels derived from an individual action (refuse vaccination). Such consequences would be difficult to identify from a human-centered vision. Tackling health problems from the OH approach requires coordination between different social and professional sectors, including education. In fact, there are current initiatives to assess vaccination from the OH approach (19) but few of them are being developed in science and health education.

Teaching the importance of vaccines should be oriented toward empowering students to make informed decisions and take individual and collective actions (10). This is an essential part of health literacy and criticality. A high development of health literacy requires the development of CT oriented to action, which corresponds to the notion of criticality.

The CT is essential to deeply understand complex phenomena, allowing the development of an independent opinion, thus empowering students (20). As shown by Authors (blinded) (10), promoting CT skills along with knowledge about vaccines would help students to perform better decision-making and develop actions according to current scientific knowledge, avoiding pseudoscientific and non-scientific premises.

As Davies and Barnett (21) pointed out, teaching CT in higher education involves considering at least six CT dimensions: (1) core skills in critical argumentation (reasoning and inference making); (2) critical judgements; (3) CT dispositions and attitudes; (4) critical being and critical actions; (5) societal and ideology critique; (6) critical creativity or critical openness. This work is focused on the second dimension, since critical judgment is essential for a suitable decision-making, and the fourth dimension as it is the most related to criticality.

The term criticality involves CT and attends to the individual identity and the critical action dimension (21). Criticality promotion among students requires the development of complex and global views regarding an issue and we argue that OH could provide the integrated view required to effectively develop criticality regarding vaccination.

Decision-making and action development require differentiating truthful information from non-scientific ideas. For this, critical ignorance (CI) is essential (22). CI can be defined as the conscious decision about ignoring part of information deliberately by selecting and filtering information to minimize the exposure to low-quality information (5). Although CI is fundamental in the post-truth era, most of the literature on vaccines and CT are based on knowledge and evidence-based argumentation to encourage critical decision-making. Despite agreeing that some extent of fundamental knowledge is needed for CT application, it is not always possible for students to have a highly-specific knowledge on each SSI. This is something that Secondary Education teachers should take into consideration and that this design addresses.

Therefore, it is necessary to provide instruction for pre-service/in-services Secondary Education teachers about how to develop the OH approach regarding vaccination to foster CI, and Criticality among their students.

## 3 Learning environment

### 3.1 Learning objective

The objective of this design is to engage Secondary Education pre-service teachers in the practice of criticality with a focus on CI to assess information on vaccination from the OH approach. Specifically, it is aimed to:

1. Explore how OH influences the understanding of vaccination.
2. Analyze how CI is mobilized to manage information about vaccination.
3. Assess how OH and CI are articulated in the practice of criticality when assessing information on vaccination.

Objective 1 can be evaluated in Modules 1, 5; objective 2 in Modules 2, 3, 5; while objective 3 in Modules 4, 5.

### 3.2 Participants

This proposal is designed to be implemented with Secondary Education pre-service teachers with a scientific background (e.g., a degree in Biology, Geology, Chemistry, Physics, Pharmacy …) who are doing a master’s degree in science education in which health controversies are addressed as part of their training in socio-scientific instruction. These pre-service teachers do not have previous experience in the classroom and this training is the first contact with science education topics and vaccination from the OH approach.

The design will be implemented within the subject “Didactic Designs on Science Education” during the next school year 2024–2025. Ethical considerations will be contemplated during this process of implementation and data analysis according to current legislation.

### 3.3 Design principles

Most of the learning environments and designs proposed in the literature to foster CT are mainly focused on the use of SSIs as a context to promote critical argumentation, due to their complex and controversial nature (23, 24). SSIs related to biology and environmental education, such as vaccination, are considered privileged contexts to foster CT development (25). These designs are mainly based on the CT framework proposed by the Delphi study of Facione (26) and in the notion of CT provided by Kuhn (27) that consider CT a dialogical practice. According to Facione CT involves several cognitive skills, affective dispositions and domain-specific knowledge. Facione’s framework is frequently used as an operative tool for teachers training in CT (28, 29).

Although we agree with the Facione (26) framework, current citizens are exposed to large amounts of information, some of which can constitute mis−/dis−/mal-information. Thus, teachers must encourage the skills to manage all this information to make decisions and take actions, even when there is low domain-specific knowledge of a certain SSI. This leads to the need to foster CI along with CT in science education (5, 22, 30). This design provides tools to put in practice CI in science education (Modules 2, 3).

**Figure 1:**
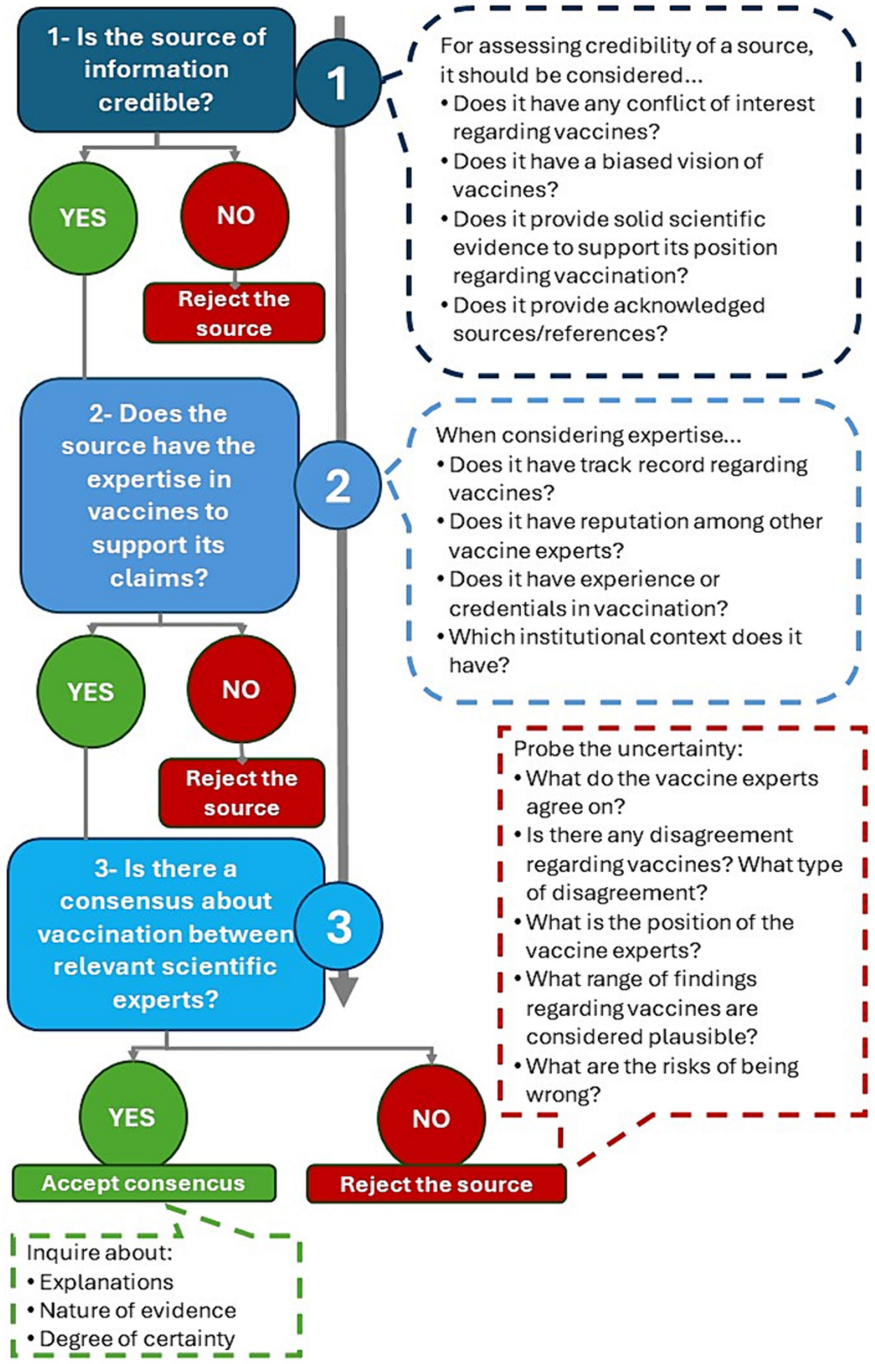
Reasoning workflow for decision-making during vaccine information evaluation [based on Osborne and Pimentel (31)].

Osborne and Pimentel (31) propose a workflow about how scientific claims and information should be evaluated. This framework is used as a framework for our design (Figure 1) (Modules 2, 3).

Our approach is focused on CI as part of CT to identify the source of information and assess its credibility as an essential dimension of CT in current societies. These are essential skills for improving decision-making, and promoting criticality (Module 4).

Developing an integral vision of vaccination is a valuable tool for criticality and vaccine promotion. We argue that OH is an approach that allows to raise awareness about different factors that affect vaccination and provides a better understanding of vaccine hesitancy. This deeper understanding of the current situation offers the chance to tackle the problem of vaccine hesitancy in a more integral way. Moreover, it allows us to use different skills of CT more efficiently when evaluating the problem. For instance, an OH approach will facilitate the application of CI when assessing information regarding vaccines. This step is essential for decision-making and taking action, when criticality becomes essential. The OH approach is included in Module 1.

## 4 Results: an instructional design for criticality on vaccination from the OH approach

Our design is a 10-h training course for pre-service/in-service Secondary Education science teachers. The instruction is organized in 5 modules (1.5 h/module). It is a flexible instruction that can be adapted to the initial level of the participants, and be scheduled according to participants’ availability. It seeks to provide efficient ways to introduce OH and Criticality to promote vaccination in science lessons. Table 1 provides an overview of the design and highlights the main dimension (OH, CI, or Criticality) addressed in each module. The OH approach underlies the whole design, as the global perspective of the problem provided by OH is necessary to properly put in practice CI and Criticality.

**Table 1:** Overview of the course for secondary education teacher instruction “How can I promote vaccination from a systemic, critical, and active perspective?”

Module	Topic/dimension		Objective	CI/OH/criticality	Duration (min)
1	How can we raise awareness about vaccination in science classrooms?		Promoting an integral vision of global risks of undervaccination.	OH	90
2	How can I help students to avoid vaccine fallacies?	(I): Looking for evidence	Providing tools for information management and to avoid over-information in relation to vaccines.	CI	45
		(II): Contrasting information			45
3	How can I raise trust in vaccines?		Developing strategies to teach about the relevance of scientific knowledge and the Nature of Science to increase trust in vaccines.	CI	90
4	What can we do to preserve public health?		Learning how to orient teaching toward critical action regarding vaccination.	Criticality	90
5	Propose a new design using OH approach		Applying the learnt during the instruction to your own classroom and context.	OH CI Criticality	90

The workflow of this design was elaborated considering the main abilities needed in different moments of information management that lead to decision-making and taking actions. Firstly, developing a wide and complex understanding of the problem is desirable. This is addressed in Module 1 where OH can be a beneficial approach to this purpose. Afterwards, this multi-step process involves managing information efficiently where CI plays a central role and that is tackled in Modules 2 and 3. The previous steps would lead to decision-making and developing actions to face the problem, when Criticality gains prominence.

### 4.1 Module 1: how can we raise awareness on vaccination?

This module includes a brainstorming and one activity to help participants understand the role of vaccines in public health and the consequences of low-vaccination coverage. Its main goal is to introduce the OH approach in connection with the problem of vaccination. Also, attention is on the way teachers can raise awareness among the students about the social problem of low vaccination coverage. For this, the concept of OH is introduced and explained.

#### 4.1.1 Activity – brainstorming

Pre-service teachers are asked to express their own opinion about health and vaccination. This will allow us to introduce the topic and also to identify their initial view. The following questions can be used to guide this activity:

- What is health? How would you define it using your own words?
- Do you think that human health can be affected by environmental and animal factors?
- If so, how do they affect human health?

#### 4.1.2 Activity – using the OH approach for assessing vaccination

Teachers are asked to apply the OH approach to the problem of undervaccination and to represent their view in a diagram. We suggest presenting the problem of infectious diseases and undervaccination as a global health problem by using the OH approach. This approach puts into perspective the complexity of new infectious-disease emergence, and the preventable-disease outbreaks. This context allows to highlight the relevance of vaccination as a community tool to prevent infectious diseases. A guiding question can be used to introduce this issue.

**Figure 2:**
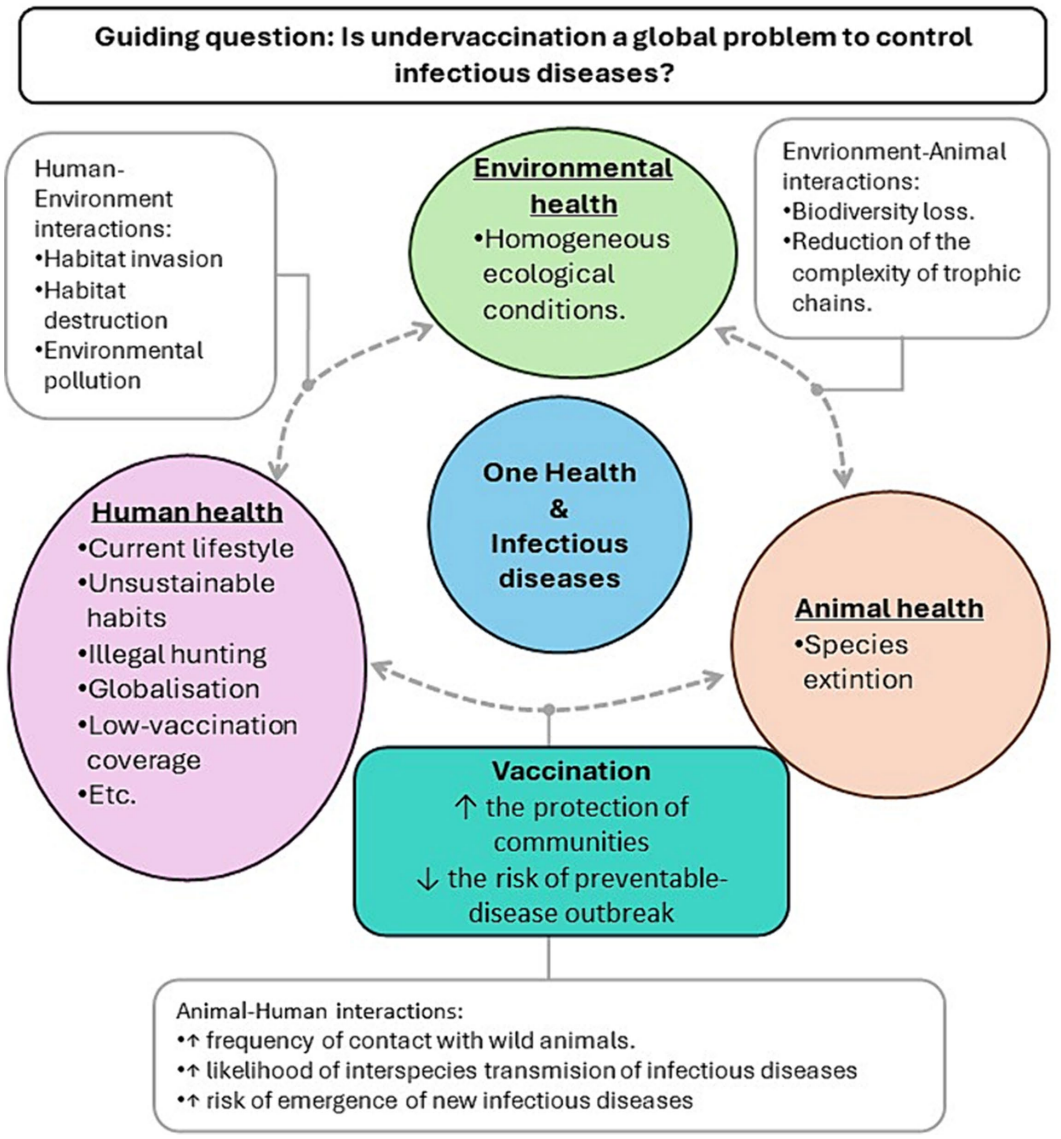
Illustrative example of the relevance of vaccination to prevent the emergence of infectious diseases from the One Health approach.

Figure 2 is an example of how OH provides a global view about infectious diseases and the role of vaccination. As showed in Figure 2 vaccination plays an important role in the system of interactions. Vaccinating domestic animals and human communities can potentially limit the risk of emergence of new infectious diseases, and specially protects the population from severe diseases when infection occurs. Hence, it mainly acts in the Animal-Human interactions.

Additionally, human factors (Figure 2, purple bubble) can also be modified to increase vaccination. At this level, vaccine education will raise awareness about the importance of a high vaccine coverage as a community “shield” against infectious diseases.

Secondly, a group discussion will be performed. Participants are asked to explain their OH approach to the rest of the group. All models are discussed among the whole group to enrich the learning. Both activities show the utility of the OH approach, and provide participants with ways to introduce the OH in their lessons.

At the end of this module it would be expected that teachers improve their ability to:

- Foster OH regarding vaccination among students.
- Raise awareness about the risks of low vaccination rates among students.
- Emphasize the importance of vaccination for maintaining a good global health status among students.

### 4.2 Module 2: how can vaccine fallacies be prevented among students? (I) (II)

After learning about the potential of OH to promote a systemic understanding of vaccination, participants are introduced into the second module, which is focused on promoting CI to avoid fallacies and reduce vaccine hesitancy when looking for information. This module consists of two sections that correspond to the first and second steps of the methodological approach (Figure 1).

To develop an opinion about vaccines, students should look for information about their utility. However, some pieces of information can be misleading, especially in the post-truth era. Thus, teachers should provide their students with tools to manage all the information they are exposed to, and CI plays a central role at this stage. Thus, the two sections of this module are focused on different steps of putting CI during the analysis of a piece of information.

#### 4.2.1 Part (I): looking for evidence

This section includes an activity focused on assessing how to tackle the first question of the methodological approach: “Is the source of information credible?”

A short overview of the process of evaluating vaccine-related information (Figure 1) is performed as this is a useful way to teach students how to manage vaccine over-information and misleading information. For a better understanding of this workflow, illustrative examples that participants must solve are provided.

##### 4.2.1.1 Activity – looking for evidence

**Figure 3:**
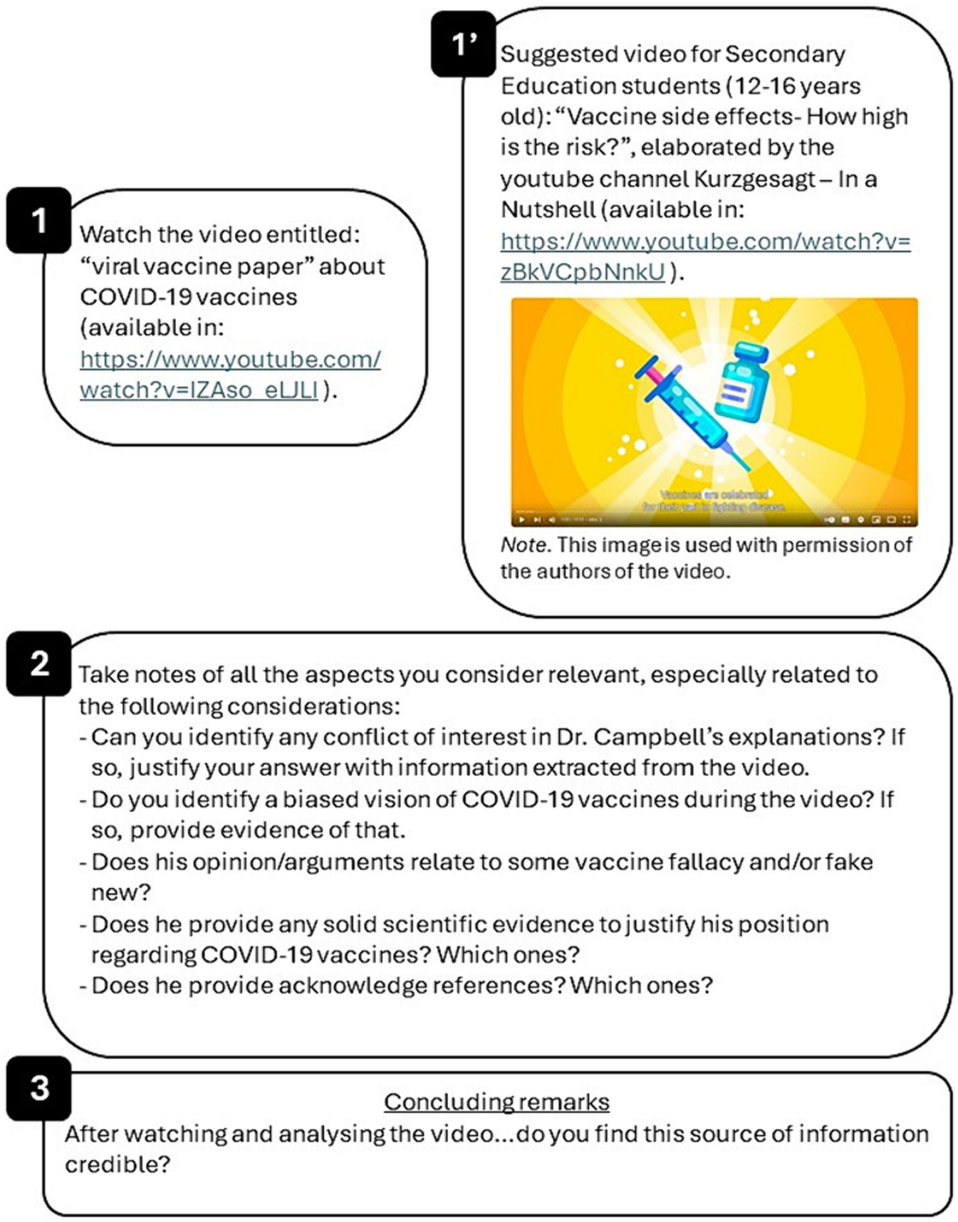
Representative example of the material provided for activity 2 “Looking for evidence.” Panel 1 includes the piece of information (in this case, a YouTube video). Panel 2 corresponds to the guiding questions used to assess the credibility of the information source. Panel 3 represents the concluding remarks that should be extracted after analyzing the questions of panel 2. Panel 1′ is an example of a piece of information adapted for developing this activity with Secondary Education students (12–16 years old) in science lessons; screenshot image from https://www.youtube.com/watch?v=zBkVCpbNnkU, © Kurzgesagt – In a Nutshell, used with permission.

Different pieces of information obtained from different sources and/or self-elaborated (i.e., news, comments on the web, videos, scientific papers…) are provided. To answer the question “Is the source of information credible?” guiding questions are also provided (Figure 3).

Note that this is adapted to the level of expertise expected from the participants (pre-service/in-service Secondary Education teachers with a biology/nature sciences/biomedical degree, or similar). However, guiding questions can be applied to any piece of vaccine information and to different levels of expertise of students (Figure 3), and so does the rest of the following activities.

At the end of this section, it would be expected teachers to improve their ability to:

- Help students to look for evidence when evaluating a piece of information to avoid vaccine fallacies.

#### 4.2.2 Part (II): contrasting information

Participants will assess the second question included in the design (Figure 1) “Does the source have the expertise in vaccines to support its claims?.” This section includes an activity focused on the expertise of the source/author of a piece of information to accept/reject a source as trustable.

##### 4.2.2.1 Activity – is expertise a criterion for credibility?

**Figure 4:**
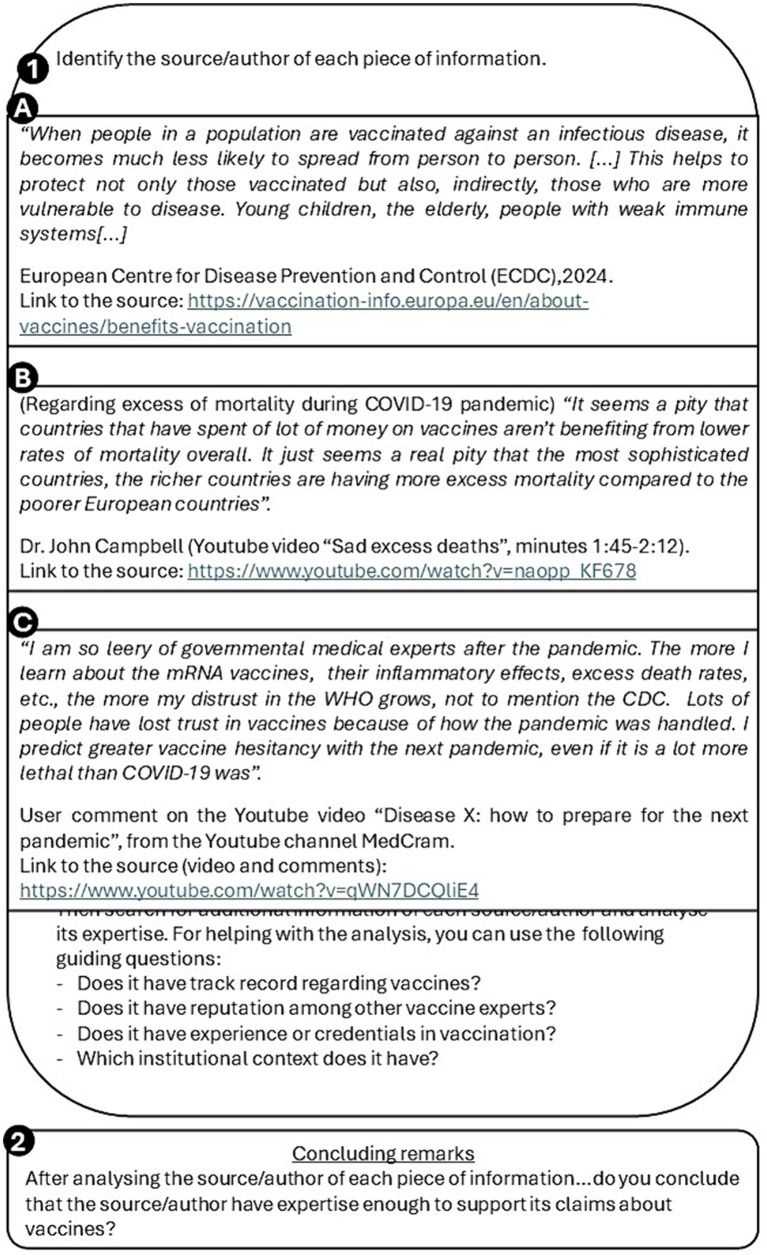
Examples of different pieces of information provided for activity 3 “Is expertise a criterion for credibility?.” Panel 1 includes different texts, whose author/source has a level of vaccine expertise that participants should evaluate using the guiding questions below the texts. Panel 2 corresponds to the concluding remarks in regard to the author/source expertise after the analysis of information in panel 1.

Different sources of information are provided for their analysis. Participants are required to look for additional information related to the source/author of each piece of information. The additional information allows them to evaluate the level of expertise of each source. Guiding questions will be included to guide the analysis (Figure 4).

The pieces of information selected for this activity (Figure 4) represent different levels of expertise and come from different types of sources. Text A is part of the website of the European Information Vaccination Portal. It can be seen that it is an official institution website of the European Centre for Disease Prevention and Control (ECDC), in partnership with the European Commission and the European Medicines Agency (EMA). It uses official data, and they intend to provide accessible and reliable information about vaccines and their surveillance. Therefore, it should be considered as a source with suitable expertise to be considered on this matter.

Text B may appear as a well-established scientific claim made by a professional. However, when additional information about the author is considered, the author happens to be a non-trustable source of information since he has a reputation of repeatedly misleading information about different topics. In fact, the claim of text B was proven false by a science fact-check website (32).

Text C is a comment from an anonymous user in a YouTube video. Since there is no possibility to assess his/her expertise, it will not be considered as a trusting source of information.

A high expertise in vaccines should be considered as a positive indicator to accept the information of the source as reliable. Nevertheless, this step should not be considered as the only indicator of reliability when assessing scientific information, as it can lead to accepting a source that is not truly evidence-based. This indicator should be considered along with the analysis performed in previous steps. An example of this is the case of the virologist Luc Montagnier, who won the Nobel Prize in Physiology or Medicine (2008) for the co-discovery of Human Immunodeficiency Virus. Taking into consideration Luc Montagnier’s wide expertise in infectious diseases, we could find all of his claims as reliable. However, he is also well known for defending homeopathy, an alternative therapy with no current scientific evidence, and for opposing vaccination, especially regarding COVID-19 vaccines (33).

At the end of the section it would be expected teachers to improve their ability to:

- Provide efficient strategies to guide students in the process of contrasting information to avoid vaccine fallacies.

### 4.3 Module 3: how can trust in vaccines be raised?

This module includes an activity to assess the third question of the design tool (Figure 1) “is there a consensus about vaccination between relevant scientific experts?,” tackling trust in science and consensus reached by the scientific community.

Teachers are aware that the pro-vaccination position of the scientific community is based on a wide range of evidence. However, vaccine hesitancy increases when the public experiences first-hand the construction of scientific knowledge, like happened during the development of COVID-19 vaccines. In this situation, vaccine education plays a central role.

#### 4.3.1 Activity – what do we currently know about vaccines?

Teachers should try to inform about the status of scientific knowledge construction regarding vaccination. This means that they should make emphasis on the process of scientific knowledge construction rather than on the results. This is vital when students are dealing with an SSI as vaccination.

Participants are divided into small groups (3–4 participants/group). Different pieces of information like the examples above mentioned (Figures 3, 4) are provided. They are asked to think about the following aspects of vaccination when dealing with vaccine-related information:

1. Do the experts agree on the safety of vaccines?
2. Do the experts agree on the efficacy of vaccines?
3. What is the position of scientific experts regarding vaccination?
4. Is there any disagreement in the scientific community regarding vaccines? If it is the case, what type of disagreement?
5. What is the evidence that supports pro-vaccination claims?
6. What is the risk of being wrong when making decisions about vaccination?

These questions will lead participants to accept the piece of information when it agrees with the predominant position of the scientific community, or to reject it when it is not aligned with the scientific community position on vaccines.

At the end of the session, we would expect teachers to improve their ability to:

- Effectively communicate the uncertainty as something inherently associated with the construction of scientific knowledge.
- Teach that science-in-the-making involves discussing positions/options based on evidence and that discrepancies can enrich this process.
- Effectively transmit the idea that when the scientific community reaches a general agreement it is supported by a well-established set of evidence.

### 4.4 Module 4: what can be done to preserve public health?

Promoting a systemic OH view of the role of vaccines (Module 1) and fostering skills related to CI when evaluating vaccine information (Modules 2, 3) can enable the empowerment of students during decision-making. Decision-making is essential to engaging them in the practice of Criticality. Criticality is the focus of this module, which includes an activity oriented toward developing actions based on the evidence and the information analyzed in the prior modules.

#### 4.4.1 Activity – action!

In this activity participants should use the OH models elaborated in 1.1/1.2 to think about the risks and benefits of a high-vaccinated population, and a low-vaccine coverage. The vaccination OH models will help them to solve a case in which they would act as the parent/family of a newborn and must decide whether or not to vaccinate their child with the new vaccine for preventing the respiratory syncytial virus (RSV). The case is discussed in pairs and each participant is assigned to a pro−/anti-vaccination position. The following questions should be answered and justified:

(Both) What are the reasons for (not to) vaccinate your child?
(Both) How would you protect your kid from RSV and other infectious diseases?
(Only “anti-”) What would you do if RSV vaccination is compulsory for accessing kindergarten education?
(Only “pro-”) What would you do if there is any RSV unvaccinated child in the kindergarten?

At the end of the session, we would expect teachers to be able to:

- Encourage students to suggest individual and collective actions to vaccinate.
- Help students to come up with OH actions, that means proposing actions related to animal health, ecological actions, human health regarding vaccination.

### 4.5 Module 5: propose a new design using OH approach

Participants are required to put in practice their abilities to foster Criticality and CI by using the OH approach. For this, they are asked to design their own learning situation using the approach proposed in this work. The output will be a table similar to Table 1. Besides, this module is used to solve doubts and apply the learned in the previous modules into participants’ real contexts in science lessons.

For elaborating their own design participants can use the following steps as a guide:

- Select an educational level, and a specific subject. This is essential to adapt the learning situation to the target group you would like to teach.
- Select the SSI topic. The context selected for introducing the SSI should be a relevant one to engage and motivate students during the teaching-learning process.
- When teaching the topic in science lessons, pay attention to the following dimensions of the SSI:
  - Social relevance of the SSI (using the OH approach).
  - SSI current situation (using the OH approach).
  - Introduce the main misconceptions related to the selected SSI.
  - Promote trust in science.
  - Make students aware of the uncertainty inherent to science-in-the-making.
- Use the questions included in the workflow of Figure 1 as a guide to teach students about how to manage information regarding the SSI through the practice of CT and CI.
- Orient the SSI learning to criticality. Make students aware of their ability to develop individual actions and to participate in collective actions.
- Formulate appropriate guiding questions to help students during the assessment of all the dimensions mentioned above.
- Provide strategies and instruments for the evaluation of the design.

At the end of the session, we would expect teachers to be able to:

- Design an original learning situation to foster vaccination from OH using CT and CI.
- Apply the workflow presented in the activities (Figure 1) to design a new learning situation for the practice of criticality.
- Apply this knowledge to other complex SSIs.

This module also includes a final questionnaire (Supplementary Figure S1) that participants should fill and that will be helpful to assess how useful this instruction is.

### 4.6 Evaluation of the design

The initial brainstorming developed in Module 1 will be used as an initial evaluation to identify the starting point of the participants. To evaluate the initial view of the participants a rubric for the questions included in the brainstorming will be used (Supplementary Table S1).

The performance of the participants during the activities will also be considered for evaluating the utility of this design. The written diagrams elaborated individually in Module 1 will give us information about their understanding of OH approach in relation to vaccines; the analysis of a piece of information in Module 2 will allow to assess if the participants are able to apply skills related to CI …This analysis will allow to introduce modifications in the design to improve its utility.

The final evaluation will be performed by analyzing the productions elaborated in Module 5. The design of their own activities will show an improvement in their ability to introduce criticality paying attention to CI regarding vaccination using the OH approach, thus helping them to achieve the learning objective. Supplementary Table S2 shows the evaluation instrument to analyze participants’ design of Module 5.

The final evaluation will also include a questionnaire to know the opinion of the participants about the utility for the instruction, shown in Supplementary Figure S1.

## 5 Discussion and practical implications

To our knowledge this is an original contribution to prepare Secondary Education teachers to address vaccination in science instruction using OH and engaging students in the practice of criticality.

This is an original design developed based on previous research about the practice of CT (21) and the model of OH (7) that uses as design principles the ones provided by Osborne’s and Pimentels’ (31) framework.

Its implementation will take place in teachers training on SSIs for Secondary Education (12–16 years old) during the second semester of 2024–2025 as part of the master’s degree subject “Didactic Designs on Science Education” at the University X (blinded). We expect that data from implementation would allow to expand the knowledge on how to promote CT and OH view in a controversial context as vaccination, and to check the adequacy of the design and the principles that oriented it. Data collection during the implementation of the activities will be oriented to this purpose, as well as to explore pre-service teachers’ performance and their understanding of the activities. This will be relevant to introduce improvements in our design.

Teaching strategies for information management are essential to avoid vaccine misconceptions and to promote vaccination. Until recent years, CT research in science education has been concerned with promoting the development of this competence among students through argumentation, the use of evidence, critical reading of texts, etc.

However, the situation in science lesson has changed considerably. The digital revolution has transformed the way we relate to each other and inform ourselves, with social networks being the main information source and exchange of ideas space. The spread of fake news is neither a new phenomenon nor characteristic of the current digital era, but the development of new technologies has facilitated its expansion and magnified its scope. CT teaching in science lessons is more difficult due to dis−/mis−/mal−/over-information as they require a greater emphasis on the development of meta-reflection, and CI. Promoting CT among students involves being able to control their own information environment rather than involving them in the process of assessing information in a way that does not allow them to ignore low-quality information (5). This design seeks to equip to teachers to adapt their lessons to the current and future challenges where tackling SSIs require an integral understanding of the problem and also CI to ignore fake news and pseudo-scientific statements.

## Data availability statement

The original contributions presented in the study are included in the article/Supplementary material, further inquiries can be directed to the corresponding author.

## Ethics statement

The studies involving human participants were reviewed and approved by the research ethics committee of the Universidade de Santiago de Compostela (USC) (Spain) (code: USC 41/2024). Written informed consent to participate in this study will be obtained from participants prior to the commencement of the study.

## Author contributions

IM-P: Writing – review & editing, Writing – original draft, Supervision, Methodology, Investigation, Conceptualization. BP: Writing – review & editing, Writing – original draft, Supervision, Conceptualization. AU: Writing – review & editing, Supervision, Conceptualization.

## References

[1] Thèves C Crubézy E Biagini P. History of smallpox and its spread in human populations. Microbiol Spectr. (2016) 4:4. doi: 10.1128/microbiolspec.PoH-0004-2014 27726788

[2] Walzer P Estève C Barben J Menu D Cuenot C Manckoundia P . Impact of influenza vaccination on mortality in the oldest old: a propensity score-matched cohort study. Vaccine. (2020) 8:3. doi: 10.3390/vaccines8030356 PMC7564344 32635210

[3] World Health Organization . COVID-19 vaccinations have saved more than 1.4 million lives in the WHO European region, a new study finds. (2024). Available at: https://www.who.int/europe/news/item/16-01-2024-covid-19-vaccinations-have-saved-more-than-1.4-million-lives-in-the-who-european-region--a-new-study-finds

[4] Larson HJ . The biggest pandemic risk? Viral misinformation. Nature. (2018) 562:309. doi: 10.1038/d41586-018-07034-4 30327527

[5] Kozyreva A Wineburg S Lewandowsky S Hertwig R. Critical ignoring as a Core competence for digital citizens. Curr Dir Psychol Sci. (2022) 32:1. doi: 10.1177/09637214221121570 PMC7615324 37994317

[6] Carolan K Verran J Crossley M Redfern J Whitton N Amos M. Impact of educational interventions on adolescent attitudes and knowledge regarding vaccination: a pilot study. PLoS One. (2018) 13:1. doi: 10.1371/journal.pone.0190984 PMC5774691 29351325

[7] Food and Agriculture Organization of the United Nations; World Organisation for Animal Health and World Health Organization . Taking a multisectoral one health approach: A tripartite guide to addressing zoonotic diseases in countries. (2019). Available at: https://www.who.int/publications/i/item/9789241514934

[8] World Health Organization, Strategic Advisory Group of Experts on Immnunization . Meeting of the Strategic Advisory Group of Experts on Immunization, October 2014: conclusions and recommendations. (2014). Available at: https://www.who.int/publications/i/item/WER8950

[9] Kennedy J . Vaccine hesitancy: a growing concern. Pediatr Drugs. (2020) 22:105–11. doi: 10.1007/s40272-020-00385-4 32072472

[10] Puig B Ageitos N “Critical Thinking to Decide what to Believe and what to Do Regarding Vaccination in Schools. A Case Study with Primary Pre-service Teachers”. In Puig B Jiménez-Aleixandre MP editors. Critical thinking in Biology and Environmental Education. Facing challenges in a post-truth world. Springer (2022), p. 113–132.

[11] Stolle LB Nalamasu R Pergolizzi JV Varrassi G Magnusson P LeQuang J . Fact vs. fallacy: the anti-vaccine discussion reloaded. Adv Ther. (2020) 37:4481–90. doi: 10.1007/s12325-020-01502-y 32965654 PMC7509825

[12] Hitti FL Weissman D. Debunking mRNA vaccine misconceptions—an overview for medical professionals. Am J Med. (2021) 134:6. doi: 10.1016/j.amjmed.2021.02.004 PMC7956899 33737059

[13] Wilson SL Wiysonge C. Social media and vaccine hesitancy. BMJ Glob Health. (2020) 5:e004206. doi: 10.1136/bmjgh-2020-004206, PMID: 33097547 PMC7590343

[14] Gardner L Dong E Khan K Sarkar S. Persistence of US measles risk due to vaccine hesitancy and outbreaks abroad. Lancet Infect Dis. (2020) 20:10. doi: 10.1016/S1473-3099(20)30522-3 32738934 PMC7392555

[15] Thompson S Meyer JC Burnett RJ Campbell SM. Mitigating vaccine hesitancy and building trust to prevent future measles outbreaks in England. Vaccine. (2023) 11:288. doi: 10.3390/vaccines11020288 PMC9962700 36851166

[16] Humer E Jesser A Plener PL Probst T Pieh C. Education level and COVID-19 vaccination willingness in adolescents. Eur Child Adolesc Psychiatry. (2023) 32:3. doi: 10.1007/s00787-021-01878-4 PMC8456192 34550459

[17] Khairat S Zou B Adler-Milstein J. Factors and reasons associated with low COVID-19 vaccine uptake among highly hesitant communities in the US. Am J Infect Control. (2022) 50:3. doi: 10.1016/j.ajic.2021.12.013 34995722 PMC8730806

[18] Hobusch U Scheuch M Heuckmann B Hodžić A Hobusch GM Rammel C . One health education Nexus: enhancing synergy among science-, school-, and teacher education beyond academic silos. Front Public Health. (2024) 11:11. doi: 10.3389/fpubh.2023.1337748 PMC10995387 38585291

[19] FAO; UNEP; WHO; WOAH ; One Health Joint Plan of Action, 2022–2026. One health joint plan of action, 2022–2026. (2022). Available at: http://www.fao.org/documents/card/en/c/cc2289en (accessed March 22, 2024).

[20] Hooks B . Teaching critical thinking. New York: Routledge (2010).

[21] Davies M Barnett R. The Palgrave handbook of critical thinking in higher education. New York: Palgrave Macmillan US (2015). 8 p.

[22] Wineburg S. To navigate the dangers of the web, you need critical thinking– But also critical ignoring. (2021). Available at: (https://theconversation.com/to-navigate-the-dangers-of-the-web-you-need-critical-thinking-but-also-critical-ignoring-158617).

[23] Sadler TD . Socio-scientific issues in the classroom. Netherlands: Dordrecht Springer (2011).

[24] Evagorou M Nielsen JA Dillon J. Science teacher education for responsible citizenship Springer Cham (2020).

[25] Puig B Jiménez-Aleixandre MP. Critical Thinking in Biology and Environmental Education. Facing challenges in a post-truth era. Cham: Springer (2022). p. 5.

[26] Facione PA . Critical thinking: a statement of expert consensus for purposes of educational assessment and instruction. (1990). Available at: https://eric.ed.gov/?id=ED315423

[27] Kuhn D . Critical thinking as discourse. Hum Dev. (2019) 62:146–64. doi: 10.1159/000500171

[28] Dominguez C . The CRITHINKEDU European course on critical thinking education for university teachers: From conception to delivery. Vila Real: UTAD (2018).

[29] Delen I . Designed based pedagogical content knowledge across European teacher education Programms. Ankara: Anı Yayıncılık (2021).

[30] Caulfield M Wineburg S. How to think straight, get duped less, and make better decisions about what to believe online. Chicago and London: The University of Chicago Press (2023).

[31] Osborne J Pimentel D. Science, misinformation, and the role of education. Science. (2022) 378:6617. doi: 10.1126/science.abq8093 36264815

[32] Health Feedback . COVID-19 vaccines aren’t associated with an increase in excess deaths, contrary to claim by John Campbell. (2023). Available at: https://healthfeedback.org/claimreview/covid19-vaccines-arent-associated-with-increase-excess-deaths-contrary-claim-john-campbell/%0A%0A (accessed March 22, 2024).

[33] Ledford H . Luc Montagnier (1932–2022). Nature. (2022) 603:223. doi: 10.1038/d41586-022-00653-y

