# Supplementary material for: One Health education for criticality on vaccination in teacher training
Source: Front Public Health. 2024 Jul 26;12:1408965. doi: 10.3389/fpubh.2024.1408965 (PMC11312376; doi:10.3389/fpubh.2024.1408965)
Supplement: Supplementary file 1 [file Data_Sheet_1.docx]

**
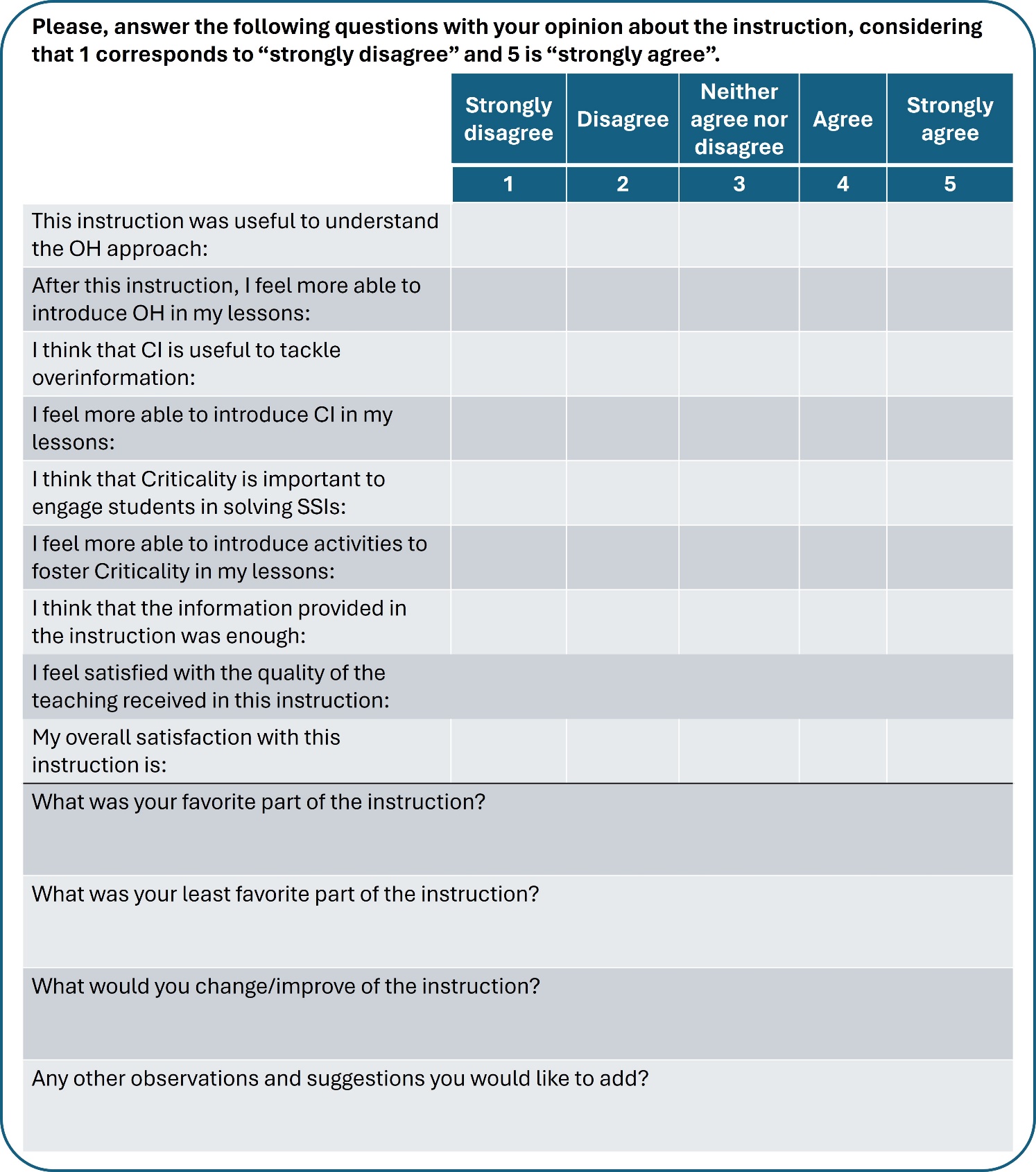
**

**Supplementary Figure S1.** Final questionnaire to evaluate the utility of the design.

**Supplementary Table S1.** Rubric for the initial evaluation of participants’ view about health, and the initial ability to identify factors that influence human health.

| **Level**  **Aspect analyzed** | **1** | **2** | **3** | **4** |
| --- | --- | --- | --- | --- |
| **Definition of health (What is health?)** | No opinion about health | Health is considered only at individual level | Health is considered both at individual and at a public level | Health is considered as a global issue that goes beyond human health |
| **Type of view about health (Do you think that human health can be affected by environmental and animal factors?)** | No health view | Human health is only affected by anthropogenic factors | Human health is affected by human, animal or environmental factors but not both (partial view) | Human health is affected by human, animal and environmental factors (OH view) |
| **Type of factors that affect health (If so, how do they affect human health?)** | No factors identified | Only human factors were identified | Human and animal or environmental factors were identified | All three types of factors (human, animal and environmental) were identified |

**Supplementary Table S2.** Rubric for the analysis of participants’ own learning situation elaborated individually in Module 5 using the OH approach to foster criticality paying attention to CI.

| **Level**  **Aspect analyzed** | **1** | **2** | **3** | **4** |
| --- | --- | --- | --- | --- |
| **Selection of the proper educational level and subject** | No educational level/subject is specified | The educational level/subject specified is not suitable for the difficulty of the design | The educational level/subject selected is suitable for the difficulty of the design | - |
| **Selection of SSI topic** | No SSI topic was selected in the design | The SSI selected is not relevant for applying this learning approach | The SSI selected is relevant for the learning approach | The SSI selected is relevant for the learning approach and a justification of the SSI selection is provided (i.e., close context for students, social relevance …) |
| **Approach selected for teaching the design: introduction of OH approach** | The design includes a human-centered vision of the issue | The design only considers the interactions human-animal or human-environment showing a partial view of the issue | The design includes an integral view of the issue, considering human, animal and environmental interactions, thus showing an OH approach | - |
| **Approach selected for teaching the design: fostering of criticality and CI** | The design does not include a description about how to implement the activities in science education | The description of the activities includes information about how to implement them, but they are not oriented towards criticality, nor CI | The description of the implementation of the activities shows that the design is oriented towards criticality and CI but no guiding questions are provided to guide students | The description of the implementation of the activities shows that the design is oriented towards criticality and CI, including guiding questions to help students during their learning process |
